# Comparative analysis of heat impacts on bond strength and failure modes of calcium silicate- and epoxy resin-based sealers

**DOI:** 10.1186/s12903-026-08520-2

**Published:** 2026-05-18

**Authors:** Nada M. Kandil, Mohamed M. Elashiry, Bryson Wong, Abeer A. Elgendy

**Affiliations:** 1 Department of Endodontics, Faculty of Dentistry, Ain Shams University, Cairo, Egypt; 2 Department of Endodontics, Dental College of Georgia, Augusta University, Georgia, USA

**Keywords:** AH Plus, Calcium silicate sealer, Cold lateral condensation, NeoSEALER Flo, Push-out bond strength, Warm vertical compaction

## Abstract

**Objectives:**

A hermetic seal is essential for successful root canal therapy. Sealers fill the interface between gutta-percha and dentin, improving adaptation and sealing ability. This study aimed to evaluate the effect of heat on the push-out bond strength and failure modes of calcium silicate- and epoxy resin-based sealers.

**Materials and methods:**

Thirty-two extracted human premolars with single straight canals were prepared and randomly assigned to two sealer groups (AH Plus, NeoSEALER Flo). Each group was further subdivided based on obturation technique: cold lateral condensation (CLC) or warm vertical compaction (WVC). Root slices from the coronal, middle, and apical thirds were subjected to a push-out test. Failure modes were classified as adhesive, cohesive, or mixed under stereomicroscopy. Data were analyzed using three-way ANOVA and multinomial mixed models with significance set at *p* < 0.05.

**Results:**

NeoSEALER Flo showed significantly higher bond strength than AH Plus across all root levels and techniques (*p* < 0.001). Heat application during WVC reduced the bond strength of AH Plus but had no effect on NeoSEALER Flo. In both sealers, the apical third exhibited the highest bond strength, followed by the middle and coronal thirds. Failure analysis revealed predominantly cohesive failures for NeoSEALER Flo, while AH Plus showed more adhesive and mixed failures under heat application.

**Conclusion:**

NeoSEALER Flo provided superior bond strength and stability regardless of obturation technique. Heat negatively influenced AH Plus but not NeoSEALER Flo, supporting the clinical advantages of calcium silicate–based sealers.

**Clinical relevance:**

The findings of this in vitro study suggest that calcium silicate-based sealers may provide improved bond strength and stability compared to epoxy resin-based sealers under different obturation techniques. However, further clinical studies are required to confirm these results under in vivo conditions.

## Introduction

The primary objective of root canal obturation is to prevent microbial recontamination following the disinfection of the canals [1, 2]. This is achieved through the utilization of gutta-percha in combination with sealing agents, which together serve to create an effective barrier that impedes the ingress of bacteria and their harmful by-products. Optimal obturation maximizes the use of gutta-percha while minimizing sealer application, ideally restricting the sealer to the periphery of the gutta-percha cone [3]. This approach enhances the apical seal, traps residual microorganisms, and limits their migration toward the periapical region, thereby improving long-term treatment outcomes [4].

Although gutta-percha is the standard obturation material, it does not adhere well to dentin and cannot effectively seal or penetrate dentinal tubules [5, 6]. Root canal sealers address this limitation by filling the space between gutta-percha and canal walls, adapting to irregularities, lateral canals, and tubules [7, 8]. Endodontic sealers vary in composition, including zinc oxide eugenol-based, calcium hydroxide-based, glass ionomer-based, silicone-based, and resin-based types [9, 10]. Epoxy resin–based sealers are widely used for their handling and physical properties [11–13], whereas calcium silicate–based sealers have become popular due to their biocompatibility, bioactivity, and effective sealing ability [14–16]. These sealers are dimensionally stable, slightly expand upon setting, and exhibit favorable properties such as thin film thickness, small particle size, high flowability, and hydrophilicity, enhancing their penetration into dentinal tubules [17, 18].

Adhesion to radicular dentin is critical to prevent fluid leakage along the sealer–dentin interface [6]. The gutta-percha material used for obturation plays a significant role in maintaining the bond between the sealer and dentin under both static and dynamic conditions. It ensures a close fit of the obturation material to the root canal dentin, which helps prevent fluid leakage when the tooth is at rest. Moreover, it can withstand mechanical forces, such as those caused by tooth flexure or post-space preparation, which could otherwise dislodge the filling material [19].

Bond strength is commonly evaluated using push-out, micro-tensile, or shear tests, with the push-out test being particularly reliable due to its ability to simulate clinical shear stresses, minimize variability, and assess different root levels in standardized 1-mm-thick slices [20–22].

This study evaluated how heat affects the bond strength and failure mode of NeoSEALER Flo compared with AH Plus. The null hypothesis was that heat application does not affect the bond strength or failure modes of either sealer.

## Materials and methods

### Ethical consideration and power analysis for sample size determination

After the approval of the Research Ethics Committee of Faculty of dentistry, Ain Shams University under the approval number (FDASU-Rec EM012388). Informed consent was taken from all patients who approved that their extracted teeth to be used in this research. This in vitro study was conducted according to the guidelines of the Declaration of Helsinki. Power analysis was designed to have adequate power to apply a statistical test of the null hypothesis that there would be no statistically significant effect of different tested variables on push-out bond strength. Using an alpha (α) level of (0.05), a beta (β) level of (0.2) corresponding to (80%) statistical power and effect size (Cohen’s f) of (0.825) derived from the results of a previous study [23]; the minimum total required sample size (n) was found to be [24] samples. The sample size was increased to account for sample variability and enhance the robustness of the statistical analysis to 32 samples (i.e., 16 samples per sealer type and 8 samples per obturation technique). The sample size was calculated using R statistical analysis software version 4.5.0 for Windows [24].

### Sample selection

Thirty-two human lower premolar teeth extracted for orthodontic reasons were collected from the Oral and Maxillofacial Surgery Department, Faculty of Dentistry, Ain Shams University. The extracted teeth were first cleaned using ultrasonic scalers and then disinfected by immersion in sodium hypochlorite (NaOCl) for 10 min. After cleaning, they were stored in a hydrated state (saline) in a closed container. All handling procedures of the extracted teeth were carried out in accordance with infection prevention and control measures. The inclusion criteria for the study were sound teeth with single, straight, and mature roots, as well as a single root canal. The average length of the included teeth was 19 ± 2 mm. Teeth with root curvature or multiple canals were excluded from the study. Additionally, teeth that exhibited caries, fractures, resorption, previous endodontic treatment, calcifications, or an initial apical size larger than a #15 endodontic file were also excluded.

### Sample classification

Teeth were categorized into two main groups based on the type of sealer used, AH Plus group and the NeoSEALER Flo group. Each sealer group was divided into two obturation technique subgroups, warm vertical compaction (WVC) with eight samples per subgroup (*n* = 8).

### Root canal treatment procedures

Radiographs were taken from both mesiodistal and buccolingual directions to confirm the presence of a single canal. The teeth were decoronated with a high-speed bur under copious water spray to standardize their length to 14 mm. The working length was determined using a #10 K‑file (MANI, Inc., Japan) inserted until visible at the apical foramen. The length was measured, and the WL was determined by subtracting 1 mm from the distance to the apical foramen. Instrumentation began with a #15 K‑file (MANI), followed by rotary instrumentation with PlexV files (Orodeka, Shandong, China) to a size #35 with a 4% constant taper, then manual instrumentation to a size 45/02. This was performed with an NSK Endomotor (Fukahodo, Kanuma, Japan) at 300 rpm and a torque setting of 200 gcm. During the instrumentation, the root canals were copiously irrigated with 5 ml of 5% NaOCl at each file change. A final irrigation was performed using 5 ml of 17% ethylenediaminetetraacetic acid (EDTA) for 1 min, followed by 5 ml of saline and then another 5 ml of 5% NaOCl for 1 min, concluded with a final flush of 5 ml of saline.

All root canals were irrigated using a 27-gauge needle inserted 1 mm short of the WL and were dried with multiple ProTaper paper points (Dentsply Sirona), matching the size of the master cone. AH Plus sealer was mixed in a 1:1 ratio according to the manufacturer’s instructions, while NeoSEALER Flo, which is premixed and stored in an airtight syringe, required no mixing and was ready for direct injection into the root canal.

In the CLC technique, the master cone gutta-percha (GP) size #45 and taper 2% was inserted to fit with tug back at the WL. Then, canal walls were coated with the sealer using the master GP cone. Then, the GP was introduced slowly into the root canal until it reached the WL. A #25 spreader was inserted 2–3 mm short of WL, and accessory cones were added until the spreader no longer advanced. Excess GP was sheared off by using a heated plugger and vertical compaction was performed at the orifice level. In the WVC technique, the NeoSEALER Flo (Avalon Biomed, Houston TX, USA) sealer was injected into the canal 2–3 mm short of WL. Then, the master GP cone was inserted into the canal until it reached the WL. In the AH Plus (Dentsply Maillefer, Tulsa OK, USA) group, the canal walls were coated with sealer using the GP cone, and then the GP was inserted to the full WL. The fast-pack unit (Eighteeth Medical Technology Co., Ltd., Changzhou, China) of the obturation system was then used for both groups at 180 °C to remove the GP 4 mm short of the WL, followed by condensation with a pre-fitted hand plugger. Backfilling was performed with the fast-fill unit (Eighteeth) at 200 °C, followed by condensation with a larger-size plugger.

Radiographs of the samples were taken to confirm the quality of the root canal filling. Samples with inadequate obturation were discarded and replaced by others. All specimens were stored for 2 weeks at 37℃ in 100% humidity to allow the sealers to set.

### Testing and evaluation

All the specimens from each of the four groups were completely embedded in clear acrylic blocks. Each root was horizontally sectioned into approximately 2 ± 0.1 mm thick slices with a low-speed IsoMet 4000 saw (0.3 mm blade) operating at 2400 rpm under continuous water refrigeration. Overall, three slices per specimen, one each from coronal, middle, and apical thirds, were obtained. Apical 3 mm from each root was discarded to avoid possible apical ramifications. Slices with defective obturation were discarded and replaced by another ones, each slice was marked from the apical surface for ease of placement during push-out test, then the apical and coronal diameter of the canal for each section was measured using a caliper as well as it was used to confirm the section thickness.

Each specimen was subjected to a push-out test where the punch moved in an apical to coronal direction at a crosshead speed of 0.5 mm/min and 90-degree angle using different sizes of plungers according to canal diameter varying from 0.4 mm for apical sections, 0.5 mm for middle sections and 0.6 mm for coronal sections in a universal testing machine, which resulted in the displacement of the filling material. The Universal Instron testing machine gave the debonding force for an individual specimen 88. The maximum force that dislodged the material was measured in Newton and converted to MPa using the formula: Debonding stress (MPa) = Debonding force (N)/area (mm^2^). The bonded interface area was calculated using the formula for the lateral surface area of a truncated cone:

$${\mathrm{A}}=\pi\left(\mathrm{R+r}\right)\surd\left[\left(\mathrm{R-r}\right)^2+\mathrm{h}^2\right],$$

where R is the coronal radius, r is the apical radius, and h is the thickness of the specimen. Bond strength (MPa) was calculated by dividing the dislodging force (N) by the bonded area (mm²). The apical and coronal diameters of each slice, as well as the specimen thickness, were precisely measured using a digital caliper and incorporated into the bonded area calculation to ensure accurate determination of bond strength values.

The mode of failure was analyzed by examining each de-bonded specimen under a stereomicroscope (2.0 Stereomicroscope with Image analyzer software, Microscope, Vardhan, India) at 25× magnification. Failures were classified according to Skidmore et al., as Type 1: Adhesive failure (at sealer dentin interface), Type 2: Cohesive failure (within sealer or dentin interface) and Type 3: Mixed failure.

### Statistical analysis

Continuous data are presented as mean and standard deviation values. They were checked for normality and variance homogeneity by viewing the distribution and using the Shapiro-Wilk and Levene tests, respectively. Push-out bond strength data were validated for assumptions and analyzed using a three-way mixed model ANOVA. Categorical data (failure mode) were presented as frequency (n) and percentage (%) values and analyzed using a three-way multinomial mixed model with Poisson approximation. Modeling was followed by post hoc comparisons of estimated marginal means (for continuous outcomes) and predicted probabilities (for categorical outcomes), utilizing the models’ error terms and adjusting for multiple comparisons using Tukey’s method. The significance level was set at *p* < 0.05 for all tests. Statistical analysis was performed with R statistical analysis software version 4.5.0 for Windows [24].

## Results

### Effect of heat on bond strength

**Fig. 1:**
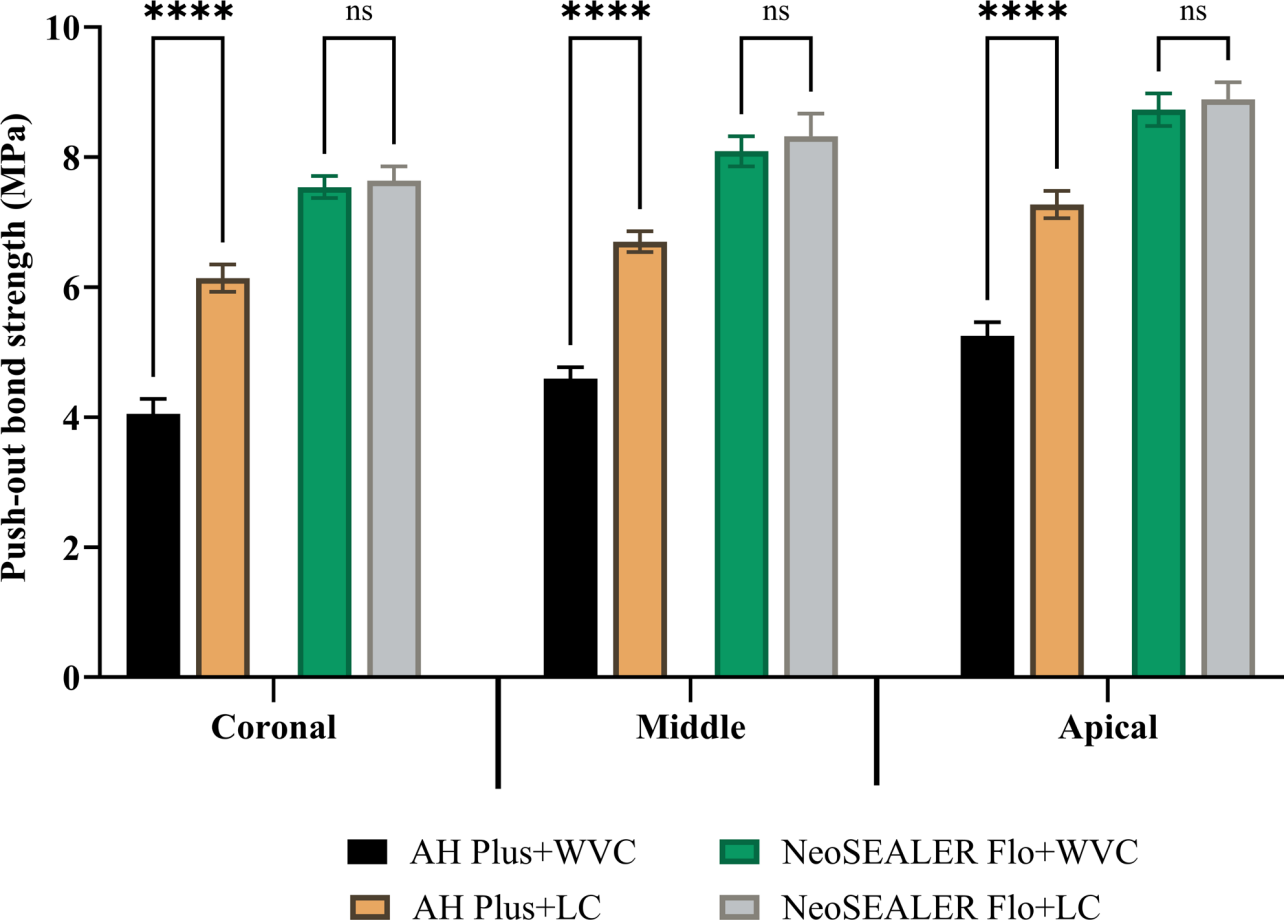
Bar chart showing mean and standard deviation values for push-out bond strength between CLC and WVC obturation techniques at the different root canal levels. ****; *p* < 0.0001, ns; non-significant

**Fig. 2:**
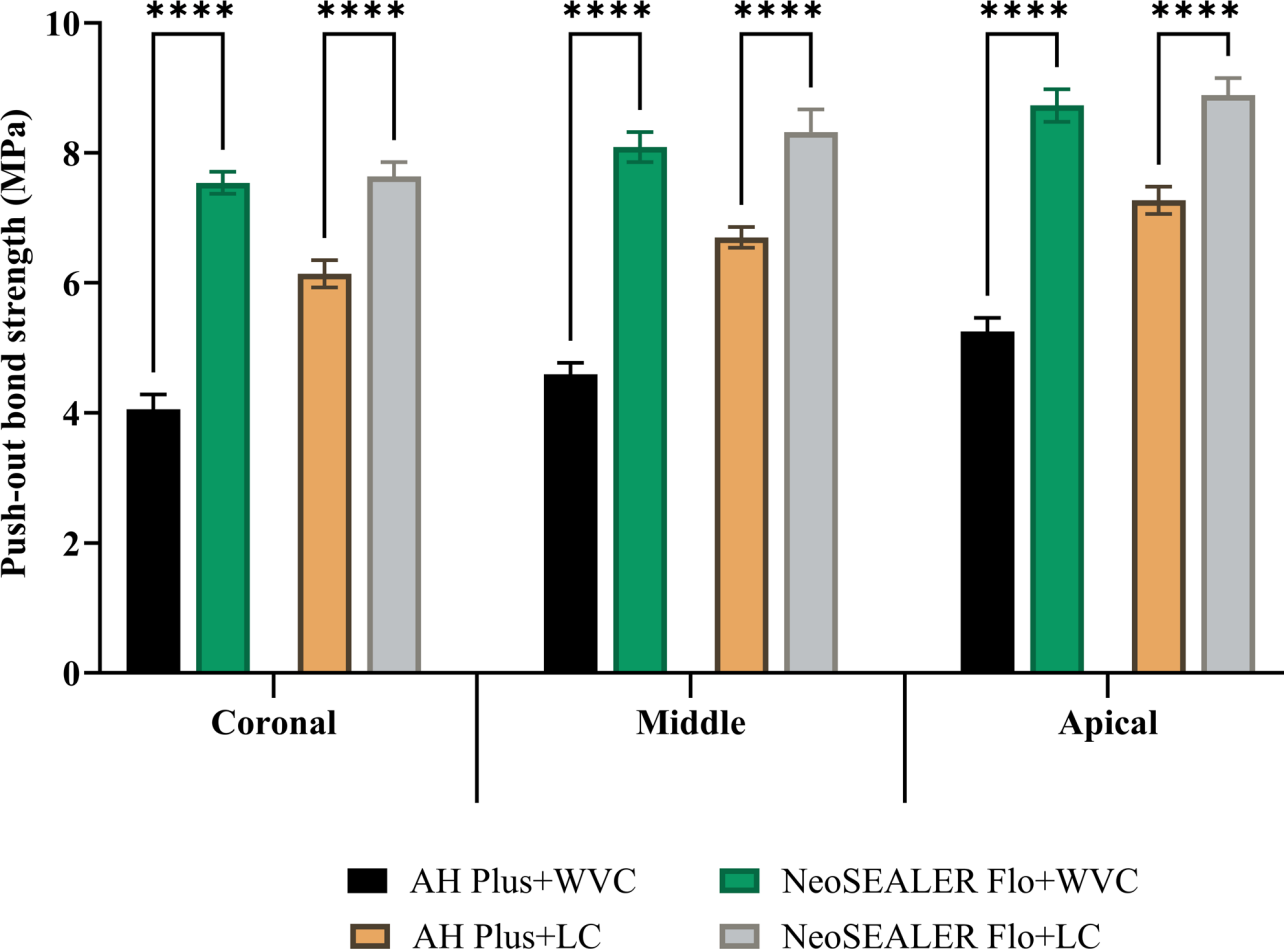
Bar chart showing mean and standard deviation values for push-out bond strength between AH Plus and NeoSEALER at the different root canal levels. ****; *p* < 0.0001

**Fig. 3:**
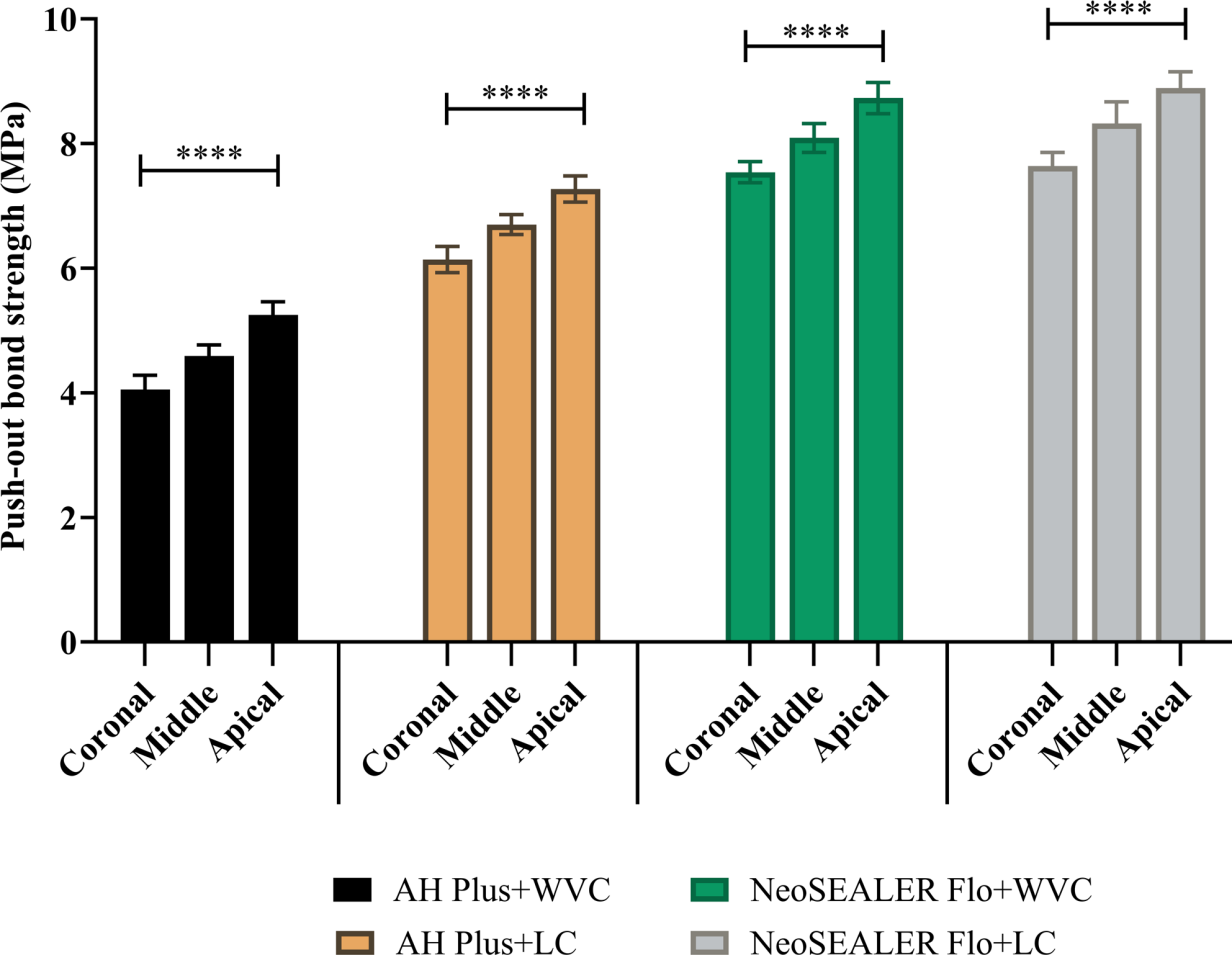
Bar chart showing mean and standard deviation values for push-out bond strength between the three root canal levels using CLC or WVC techniques. ****; *p* < 0.0001

In the AH Plus group, CLC technique displayed significantly higher bond strength than WVC at all root levels (*p* < 0.001; Fig. 1). In contrast, NeoSEALER Flo showed no significant difference between CLC and WVC (*p* > 0.05; Fig. 1). Across all levels and techniques, NeoSEALER Flo exhibited significantly higher bond strength than AH Plus (*p* < 0.001; Fig. 2). For both sealers, the apical third had the highest strength, followed by the middle and coronal thirds (*p* < 0.001; Fig. 3).

### Effect of heat on failure mode

**Fig. 4:**
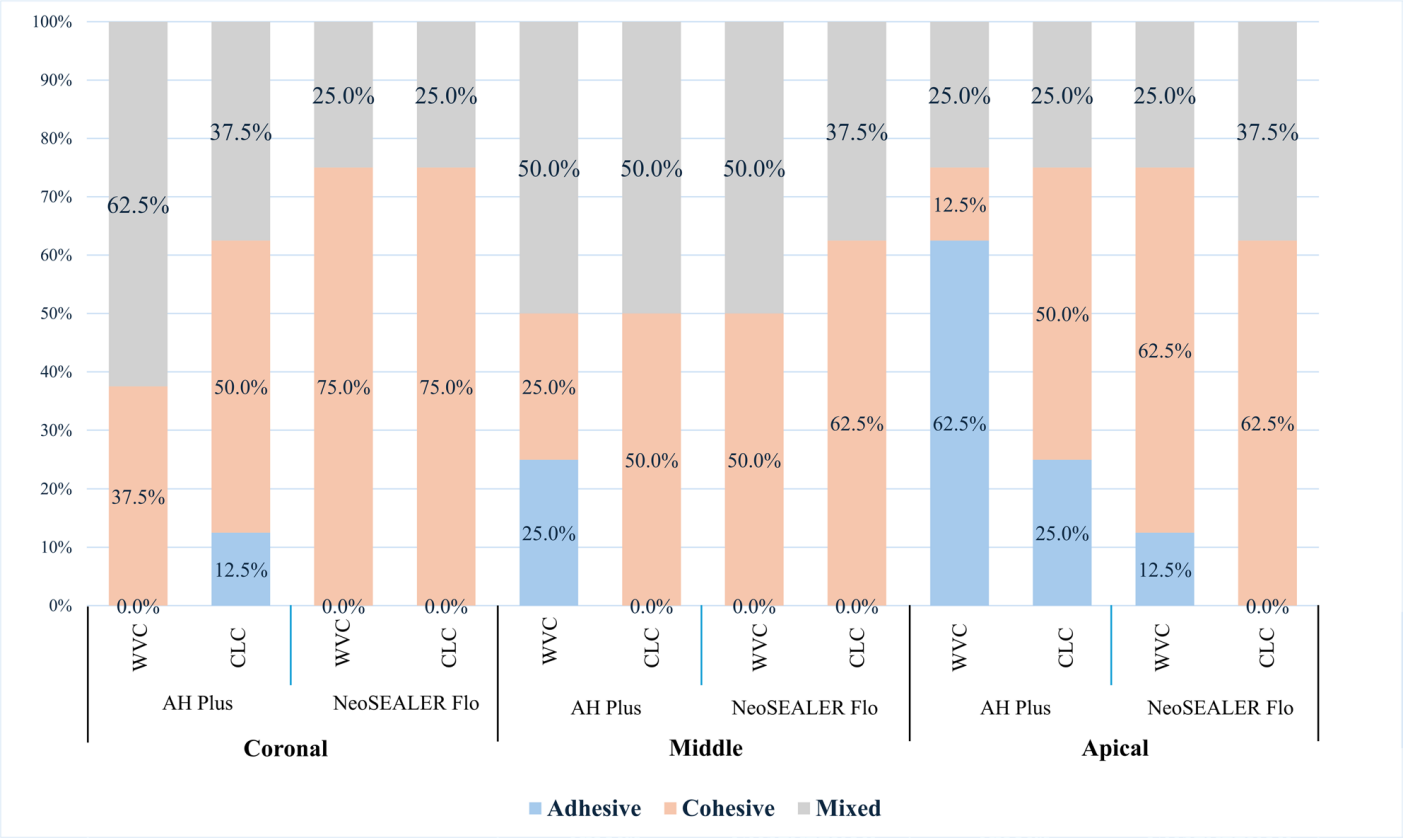
Stacked bar chart showing failure modes’ distribution between CLC and WVC obturation techniques in AH Plus and NeoSEALER at the different root canal levels

At the coronal and middle levels, AH Plus showed more mixed failures with WVC and more cohesive failures with CLC were, though differences were not significant (*p* > 0.05). At the apical level, WVC led mainly to adhesive failures, while CLC produced mostly cohesive failures (*p* > 0.05). NeoSEALER Flo predominantly exhibited cohesive failures at all levels, regardless of technique, with no significant differences (*p* > 0.05; Fig. 4).

In the comparative analysis of the modes of failure between the two sealers utilized, no significant differences were observed across any of the obturation techniques at the three levels of root canals examined (*p* > 0.05).

## Discussion

Successful endodontic treatment requires eliminating microorganisms and preventing reinfection through an effective seal [1]. Attaining a complete hermetic seal between the canal walls and the core filling material is of utmost importance for a better and more predictable outcome. Sealers play a pivotal role in this process, as gutta-percha alone does not naturally adhere to the canal walls or conform to the canal’s complex anatomy. Their primary function is to fill the void between the core filling material and the canal walls, penetrating irregularities and dentinal tubules to enhance sealing effectiveness and treatment success [7, 9].

An important indicator of a sealer’s performance is its ability to bond effectively to the root canal walls and maintain integrity under functional stresses, post space preparation, or during restoration placement [25]. This study evaluated the effect of heat during obturation on the bond strength and failure mode of the calcium silicate-based NeoSEALER Flo compared to the epoxy resin-based AH Plus.

Calcium silicate-based sealers have gained popularity due to their bioactive properties, including tissue regeneration potential, high pH, antibacterial activity, excellent biocompatibility, and dimensional stability [14, 17]. Calcium silicate-based sealers are also known for forming chemical bonds with dentin during the setting process, which produces hydroxyapatite. According to previous investigations, these sealers demonstrate exceptional stability by utilizing the moisture naturally present in the dentinal tubules to complete their setting reaction, preventing shrinkage [26, 27]. This creates a fluid-tight, gap-free seal between the obturation material and the root dentin. Furthermore, these sealers easily penetrate the dentinal tubules, contributing to the establishment of a tight seal [28].

Multiple effective methods are available for evaluating the adhesive properties of obturation materials [20]. The push‑out test was chosen for its reliability and ability to simulate clinical dislodging forces across different root levels and produces shear stresses at the sealer–dentin interface, which closely resemble the functional stresses exerted on endodontic materials in vivo [29]. Although this test does not directly predict clinical outcomes, it provides a reliable comparative measure of different sealers and obturation techniques [30].

Our results demonstrated that heat application during WVC did not affect the bond strength of NeoSEALER Flo. In contrast, heat reduced the bond strength of AH Plus, partially rejecting the null hypothesis. Prior research has consistently shown that heat exerts minimal influence on the chemical composition of both conventional calcium silicate-based sealers and their heat-resistant versions [30]. Furthermore, multiple studies have indicated that heat-based obturation techniques do not reduce the bond strength of calcium silicate-based sealers [31, 32]. While our findings align with previous studies, another study reported that heat used during thermo-plasticized injectable techniques reduced the bond strength of calcium silicate-based sealers [33]. These controversial findings highlight the need of further research in this field. The notable reduction in bond strength observed in the AH Plus group aligns with findings from previous investigations and may be attributed to the effect of heat application accelerating the chemical setting reaction of the epoxy resin-based sealer. This accelerated reaction could hinder the sealer‘s ability to adequately flow into the intricate anatomies of the root canal system, including the dentinal tubules, thereby resulting in reduced bond strength [34, 35].

Regardless of the obturation technique employed, NeoSEALER Flo consistently showed superior push-out bond strength compared to AH Plus. This performance is attributed to its physical properties, hydrophilicity, and low contact angle, which promote optimal adaptation, ion exchange, and formation of a mineral infiltration zone at the dentin interface, reducing gap formation [36–38]. The superior bond strength of calcium silicate-based sealers can be attributed to the hydration reaction of calcium silicate–based sealers, which plays a fundamental role in enhancing their adhesion to dentin. Upon contact with moisture from dentinal tubules, NeoSEALER Flo undergoes a hydration process in which tricalcium silicate reacts with water to form calcium silicate hydrate (C–S–H) gel and calcium hydroxide. The released calcium ions subsequently interact with phosphate ions present in dentinal fluid, leading to the nucleation and growth of a hydroxyapatite layer at the sealer–dentin interface. This biomineralization mechanism improves the micromechanical interlocking within dentinal tubules and promotes chemical bonding through the formation of an interfacial mineral infiltration zone. Together, these processes contribute to stronger adhesion and consequently higher push-out bond strength values compared to the non-bioactive resin-based sealer [2, 39, 40].

This finding aligns with a recent scanning electron microscopy study that showed that calcium silicate-based sealers have superior adaptability to the root canal walls compared to AH Plus sealer [38].

Our findings indicated that the bond strength is significantly greater in the apical sections compared to the middle and coronal sections for both sealers examined. This observation is consistent with previous studies, which have attributed these differences to the geometrical configurations of the root canal that enhance bond stability apically while facilitating dislodgment at the middle and the coronal Sects [41, 42]. Previous studies showed that higher bond strength was recorded in both calcium silicate-based and epoxy resin-based sealer at the apical and middle sections than the coronal Sect [43]. This may be due to a more accurate adaptation of the GP cone to the shape and size of the root canal in the middle and apical thirds which can generate higher hydraulic forces and improve the adaptation of the materials to the canal walls, resulting in a thinner sealer layer in this area [44]. Moreover, the small particle size of bio-ceramic sealers might result in improved distribution in the dentinal tubules, particularly in the smaller tubules of the apical third of the canals [2, 45]. However other studies showed contradictory results where the percentage of sealer penetration was higher at the coronal third than apically [39, 46]. These inconsistencies across studies highlight the need for cautious interpretation of the results and indicate that further research is necessary to obtain more consistent evidence.

Furthermore, the variation in push-out bond strength can largely be explained by the circular shape of the root canal in the apical area, in contrast to the typically oval, trapezoidal, or flattened shapes observed in the coronal and middle sections [42].

Failure mode analysis showed that NeoSEALER Flo predominantly failed cohesively, indicating strong bonding and adaptability unaffected by heat or canal level. Conversely, AH Plus exhibited more mixed and adhesive failures under heat during WVC but cohesive failures with CLC. Previous study has shown that sealers with high push-out bond strength, such as NeoSEALER Flo in this study, are predominantly associated with cohesive failure modes. In contrast, sealers with lower bond strength often exhibit mixed or adhesive failures [47]. Our study corroborates another investigation that reported a majority of cohesive failures occurring in calcium silicate-based sealers [48, 49]. Clinically, the superior bond strength and predominantly cohesive failure pattern observed with calcium silicate–based sealers may indicate enhanced interfacial integrity and resistance to dislodgement over time. This could potentially translate into improved long-term sealing ability and reduced risk of reinfection, particularly in cases where thermos-plasticized obturation techniques are employed [48, 49].

Overall, differences in failure mode were not statistically significant, as a result, the null hypothesis, which suggests that heat does not influence the mode of failure, was accepted. Additional research is required to identify the optimal obturation technique for each type of sealer. This will ensure the longevity and effectiveness of root canal fillings, ultimately enhancing patient outcomes and the overall success of endodontic treatments.

This study has several limitations that should be considered when interpreting the findings. First, it was conducted in vitro, which does not fully replicate the complex biological and mechanical conditions present in the oral environment; therefore, the results may not directly translate to clinical performance. Second, the use of extracted premolars with standardized canal anatomy does not reflect the natural variability encountered in clinical cases, such as curved, oval, or calcified canals. Additionally, only two types of sealers and two obturation techniques were evaluated, which limits the generalizability of the outcomes to other materials or techniques. Finally, the study assessed bond strength at a single time point and did not account for the potential effects of aging, thermal cycling, or long-term degradation, all of which may influence sealer–dentin bonding over time. Further in vivo and long-term investigations are needed to validate these findings under clinical conditions.

## Conclusion

Within the limitations of this study, calcium silicate–based sealers showed higher push‑out bond strength than AH Plus when used with either CLC or WVC. Heat application during WVC did not noticeably affect the bond strength of the calcium silicate–based sealers. However, AH Plus demonstrated lower bond strength when exposed to heat during WVC. These results highlight the importance of selecting an appropriate obturation technique when working with resin‑based sealers, as heat application may adversely affect their adhesion.

## Data Availability

The data that support the findings of this study are available from the corresponding author upon reasonable request.
